# The Transmission Properties of One-Dimensional Photonic Crystals with Gradient Materials

**DOI:** 10.3390/ma15228049

**Published:** 2022-11-14

**Authors:** Lixin Fu, Mi Lin, Zixian Liang, Qiong Wang, Yaoxian Zheng, Zhengbiao Ouyang

**Affiliations:** 1 Key Laboratory of Optoelectronic Devices and Systems of Ministry of Education and Guangdong Province, College of Physics and Optoelectronic Engineering, Shenzhen University, Shenzhen 518060, China; 2 College of Electronics and Information Engineering, Shenzhen University, Shenzhen 518060, China

**Keywords:** one-dimensional photonic crystals, gradient materials, photonic band gap, defect mode, tunable filters

## Abstract

In this paper, we studied the transmission properties, including photonic band gap (PBG) and defect mode properties, of one-dimensional photonic crystals (1D PCs) consisting of gradient materials. When keeping the average refractive index of the gradient materials in the 1D gradient-material PCs (1D GPCs) the same as the index of the corresponding normal materials in the 1D normal-material PCs (1D NPCs), by transfer matrix method, we found that the complete 1D GPCs with high-index gradient materials benefit to achieve larger omni-PBG than that in 1D NPCs. In our high-index gradient material case, for TE(TM) wave, the optimal omni-PBGs in 1D GPCs with first- and second-order gradient materials are 38.6% (50.2%) and 15.9% (22.3%) larger than that in 1D NPCs; while for the optimal relative bandwidths of omni-PBG, the corresponding promotions are 41.1% (52.3%) and 16.1% (22.6%), respectively. In addition, when defective 1D GPCs have gradient-material defect, the position of defect modes can be adjusted by selecting proper parameters of the gradient materials. These types of research are useful for designing wide PBG devices and tunable narrow-band filters which have potential application in optical communication.

## 1. Introduction

Photonic crystal (PC), firstly proposed by E. Yablonovitch and S. John in 1987 [1,2,3], is a type of novel artificial materials with periodic structure. In the past decades, PCs have been systematically studied due to their unique characteristics such as photonic band gap (PBG), localized modes, self-collimating phenomena, and surface states, which provide an effective and reliable way for the manipulation of electromagnetic (EM) wave or light [4,5,6,7]. Many devices have been fabricated based on PCs [8,9,10,11].

It is well known that complete (or perfect) PCs possess PBGs in which the propagation of EM wave in the certain range of frequency is strongly forbidden. However, if the periodicity of a complete PC is destroyed by introducing a defect, localized defect modes may appear inside the PBG [12]. The PBG effect and the localized defect mode are the most important properties of PCs, thus many PBG- and defect-mode-based devices have been designed for microwave, terahertz, or optical applications [13,14].

For example, in 2016 Yang et al. proposed a kind of one-dimensional (1D) PC nanobeam waveguides sensor array. They constructed special waveguides by assembling one central 1D defective PC and two sided asymmetric 1D complete PCs, and realized high-quality multiplexers at the wavelength of 1490, 1520, and 1550 nm [15]. In 2022, researchers from Jouf University and Beni-Suef University used 1D PCs as a sensor to detect heavy metals ions in water contamination. By detecting the change of defect modes caused due to the change in concentrations of the heavy metals in the contaminated water, they demonstrated their designs could be a potential way for simple and accurate detection of heavy metals in the contaminated water [16]. Also, some researchers used 1D PC of Si/SiO_2_ that has a wide and high reflection PBG for the detection of ethyl butanoate, which could be promising for accurate and rapid diagnosing of COVID-19 [17].

From above, we see that most of the researches mainly put their attention on the properties of PC composed of normal (or conventional) materials, i.e., the refractive indexes of the materials for PCs are constant [18,19,20]. However, the performances of the 1D PC consisting of gradient materials are quite different from that with normal materials [21,22]. Therefore, it is necessary to put some effort into the research of 1D gradient-material PCs (1D GPCs).

Recently, Singh et al. have done some researches on the 1D PCs composed of linear [23], e-exponential [24], and hyperbolic gradient materials [25]. Some interesting results were found with the presence of gradient materials. In this paper, we systematically study the properties of 1D GPCs with first- and second-order gradient materials. Different from that in Singh’s researches, when comparing the performances of 1D GPCs with the corresponding 1D normal-material PCs (1D NPCs), the average refractive index ($\bar{n}$) of the gradient materials in the 1D GPCs are always kept the same as the index of the corresponding normal materials in the 1D NPCs (the reference model). We call this a fair comparison.

In this paper, by using the transfer matrix method (TMM), the features of TE-, TM-, omni-, and complete PBG (C-PBG) in complete 1D GPCs are studied. We found that the complete 1D GPCs with high-index gradient materials benefit to achieve larger omni-PBG than that in 1D NPCs. In addition, the performances of defective 1D GPCs with gradient material defect are also studied. We found that the position of defect modes within the gap can be controlled by selecting proper parameters of the gradient materials.

## 2. Physical Model and Numerical Method

### 2.1. Physical Model

In our study, the refractive index of the gradient materials is given by:

(1) $$n_{G}=kx^{u}+C,$$

where k and u are the slope and order of the refractive index, and C is the initial refractive index. In the following, first- and second-order (u=1, 2) of $n_{G}$ are considered. It should be pointed out that, in investigations we find that for u≥3, the properties of PBGs and defect modes of 1D GPCs will change with the change of u, however, they are quite similar to that for u=2. To save space, only u=1, 2 are considered. The average refractive index of the gradient materials can be calculated by:

(2) $${\bar{n}}_{G}=\frac{1}{d_{G}}\int_{0}^{d_{G}}n_{G}dx.$$

**Figure 1:**
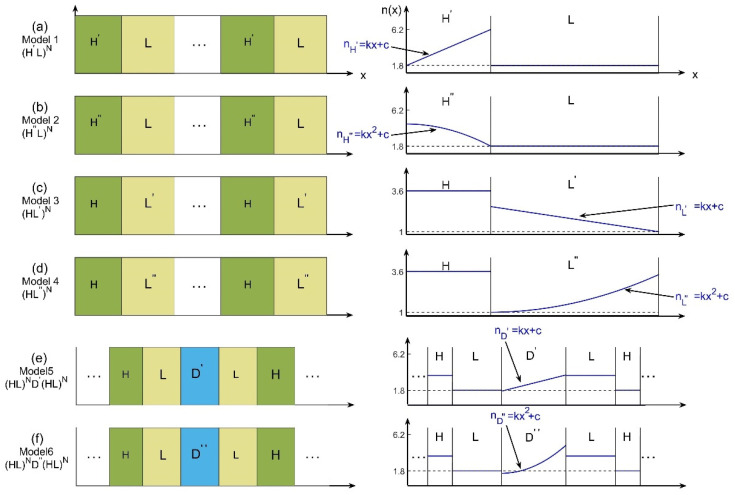
Schematics and distributions of refractive index for Models 1–6 from (**a**–**f**). Models 1–4 are the complete 1D GPCs for the research of PBG, and Models 5–6 are the defective 1D GPCs for the research of defect mode.

Here we assume that the PC structures are arranged along *x*-direction as shown in Figure 1. $d_{G}$ is the thickness of the gradient materials, so that $0\sim d_{G}$ is a complete layer of gradient material.

As illustrated in Figure 1, Models 1–4 are complete 1D GPCs for the research of PBG, and Models 5–6 are the defective 1D GPCs for the research of defect mode, where ($H^{\prime }$, $L^{\prime }$, $D^{\prime }$), ($H^{\prime \prime }$, $L^{\prime \prime }$, $D^{\prime \prime }$), and (H, L) represent the first-order (u=1) gradient-material layers, the second-order (u=2) gradient-material layers, and the normal-material layers, respectively. *N* is the number of periods.

For the research of PBG, four types of models are considered. They are, from models 1–4, corresponding to ${\left(H^{\prime }L\right)}^{N}$, ${\left(H^{\prime \prime }L\right)}^{N}$, ${\left(HL^{\prime }\right)}^{N}$, and ${\left(HL^{\prime \prime }\right)}^{N}$, respectively. In models 1 and 2, $H^{\prime }$ and $H^{\prime \prime }\;$denote the high-index gradient-material-layers, and L is the low-index normal-material-layer. Suppose $n_{H^{\prime }}$ and $n_{H^{\prime \prime }}$ are the refractive index of $H^{\prime }$ and $H^{\prime \prime }$, then they both satisfy Equation (1) for u=1, 2, respectively. According to Equation (2), the average refractive index of the gradient materials can be calculated. Here we choose ${\bar{n}}_{H^{\prime }}={\bar{n}}_{H^{\prime \prime }}=3.6$ and $n_{L}=1.8$, so it is obvious that in Models 1 and 2 ${\bar{n}}_{H^{\prime }}({\bar{n}}_{H^{\prime \prime }})>n_{L}$ are satisfied. While in Models 3 and 4, $L^{\prime }$ and $L^{\prime \prime }$ denote the low-index gradient-material layers, and H is the high-index normal-material layer. In these two cases, we choose $n_{H}=3.6$ and ${\bar{n}}_{L^{\prime }}={\bar{n}}_{L^{\prime \prime }}=1.8$ and we have $n_{H}>{\bar{n}}_{L^{\prime }}\left({\bar{n}}_{L^{\prime \prime }}\right)$.

In the PBG study, the quarter-wave stack method [26] is used to construct all the 4 Models, i.e., the optical thicknesses of them are set to satisfy ${\bar{n}}_{H^{\prime }}d_{H^{\prime }}={\bar{n}}_{H^{\prime \prime }}d_{H^{\prime \prime }}=n_{L}d_{L}=\lambda_{0}/4$ for Models 1 and 2, and $n_{H}d_{H}={\bar{n}}_{L^{\prime }}d_{L^{\prime }}={\bar{n}}_{L^{\prime \prime }}d_{L^{\prime \prime }}=\lambda_{0}/4$ for Models 3 and 4, where $\lambda_{0}$ is the character wavelength, and $d_{H^{\prime }}$, $d_{H^{\prime \prime }}$, $d_{L}$, $d_{H}$, $d_{L^{\prime }}$, $d_{L^{\prime \prime }}$ are the thicknesses of the corresponding layers. In addition, for the purpose of comparison, the performance of 1D NPC ${\left(HL\right)}^{N}$ with only normal-material layers ($n_{H}=3.6$ and $n_{L}=1.8$) are also studied. For example, in Models 1 and 2, the performances of ${\left(H^{\prime }L\right)}^{N}$, ${\left(H^{\prime \prime }L\right)}^{N}$ and ${\left(HL\right)}^{N}$ will be compared. In Models 3 and 4, the performances of ${\left(HL^{\prime }\right)}^{N}$, ${\left(HL^{\prime \prime }\right)}^{N}$ and ${\left(HL\right)}^{N}$ will be compared. We call these a fair comparison because in this way the average refractive indexes of the gradient materials in 1D GPCs are the same as the index of the corresponding normal materials in the 1D NPCs (the reference model).

For the research of defect mode, two types of models are considered. They are, from Models 5–6, corresponding to ${\left(HL\right)}^{N}D^{\prime }{\left(LH\right)}^{N}$, ${\left(HL\right)}^{N}D^{\prime \prime }{\left(LH\right)}^{N}$, respectively. In Models 5 and 6, $D^{\prime }$ and $D^{\prime \prime }$ denote the gradient-material-defects, and ${\left(HL\right)}^{N}\;$and ${\left(LH\right)}^{N}$ are the left and right repeated layers by normal materials. In the following, we select $n_{H}=3.6$, $n_{L}=1.8$ and ${\bar{n}}_{D^{\prime }}={\bar{n}}_{D^{\prime \prime }}=4.5$.

In the defect mode study, the optical thicknesses of the models are selected to satisfy $n_{H}d_{H}=n_{L}d_{L}=\lambda_{0}/4$ and ${\bar{n}}_{D^{\prime }}d_{D^{\prime }}={\bar{n}}_{D^{\prime \prime }}d_{D^{\prime \prime }}=\lambda_{0}/2$ for Models 5 and 6. Also, for comparison, the performances of structure ${\left(HL\right)}^{N}D{\left(LH\right)}^{N}$ with normal-material-defect are also studied. For example, in Models 5 and 6, the defect-mode performances of ${\left(HL\right)}^{N}D^{\prime }{\left(LH\right)}^{N}$, ${\left(HL\right)}^{N}D^{\prime \prime }{\left(LH\right)}^{N}$ will be compared with ${\left(HL\right)}^{N}D{\left(LH\right)}^{N}$, where the defect layers satisfy ${\bar{n}}_{D^{\prime }}={\bar{n}}_{D^{\prime \prime }}=n_{D}$. It is also a fair comparison because in this way the average refractive indexes of the gradient defects in 1D GPCs are the same as the index of the corresponding normal defect in the 1D NPCs.

### 2.2. Numerical Method

The TMM method is performed to study the properties of our models. Assuming the wave or light is injected from vacuum at an angle θ onto the structure in the +*x* direction. 

For the normal-material layer, the characteristic matrix can be calculated by:

(3) $$M_{N}=\left[\begin{array}{cc} cos\beta_{N} & -\frac{i}{p_{N}}sin\beta_{N}\\ -ip_{N}sin\beta_{N} & cos\beta_{N} \end{array}\right],$$

where $\beta_{N}=\left(2\pi /\lambda \right)n_{N}d_{N}cos\theta_{N}$, and $p_{N}=\sqrt{\varepsilon_{N}/\mu_{N}}cos\theta_{N}$ for TE wave, and $p_{N}=\sqrt{\mu_{N}/\varepsilon_{N}}cos\theta_{N}$ for TM wave with $d_{N}$, $n_{N}$, λ, and $\theta_{N}$ being, respectively, the geometrical thickness of the layer, the refractive index of the layer, the wavelength of the incident wave, and the angle between the wave vector and the surface normal vector of the layer.

For the gradient-material layer, the situation is different. We divide each gradient-material layer into *q* sublayers, calculate the sub-matrix of each sublayer, and multiply them together to get the characteristic matrix of one complete gradient-material layer [27]. That is:

(4) $$M_{G}=\overset{q}{\underset{j=1}{\prod }}U_{j}=\left[\begin{array}{cc} 1 & -i\frac{2\pi }{\lambda }\Psi_{2}\\ -i\frac{2\pi }{\lambda }\Psi_{1} & 1 \end{array}\right].$$

In Equation (4), $U_{j}$ is the sub-matrix of one sublayer and it fits Equation (3). When *q* is big enough (here we select *q* = 200, which is big enough for the convergence of results), the thickness of sublayers $d_{j}$ is approximate to 0. Then $\beta_{j}=\left(2\pi /\lambda \right)n_{j}d_{j}cos\theta_{j}\to 0$, $cos\beta_{j}\to 1$, and $sin\beta_{j}\to \beta_{j}$. So $U_{j}$ can be simplified to be:

(5) $$U_{j}=\left[\begin{array}{cc} 1 & -\frac{i}{p_{j}}\beta_{j}\\ -ip_{j}\beta_{j} & 1 \end{array}\right].$$

As a result, $\Psi_{1}$ and $\Psi_{2}$ can be calculated as:

(6) $$\Psi_{1}=\overset{q}{\underset{j=1}{\sum }}p_{j}n_{j}d_{j}cos\theta_{j}=\{\begin{array}{c} \overset{q}{\underset{j=1}{\sum }}\left(\varepsilon_{j}-\frac{{\left(n_{j}sin\theta_{j}\right)}^{2}}{\mu_{j}}\right)d_{j},\;\;\;\;TE\\ \overset{q}{\underset{j=1}{\sum }}\left(\mu_{j}-\frac{{\left(n_{j}sin\theta_{j}\right)}^{2}}{\varepsilon_{j}}\right)d_{j},\;\;\;\;TM \end{array}$$

(7) $$\Psi_{2}=\overset{q}{\underset{j=1}{\sum }}\frac{n_{j}}{p_{j}}d_{j}cos\theta_{j}=\{\begin{array}{c} \overset{q}{\underset{j=1}{\sum }}\mu_{j}d_{j},\;\;\;\;TE\\ \overset{q}{\underset{j=1}{\sum }}\varepsilon_{j}d_{j},\;\;\;\;TM \end{array}$$

when *q* is big enough, we can rewrite $\Psi_{1}$ and $\Psi_{2}$ with integral forms as:

(8) $$\Psi_{1}=\{\begin{array}{c} \int_{0}^{d_{G}}\left(\varepsilon_{G}-\frac{{\left(n_{G}sin\theta_{G}\right)}^{2}}{\mu_{G}}\right)dx,\;\;\;\;TE\\ \int_{0}^{d_{G}}\left(\mu_{G}-\frac{{\left(n_{G}sin\theta_{G}\right)}^{2}}{\varepsilon_{G}}\right)dx,\;\;\;\;TM \end{array}$$

(9) $$\Psi_{2}=\{\begin{array}{c} \int_{0}^{d_{G}}\mu_{G}dx,\;\;\;\;TE\\ \int_{0}^{d_{G}}\varepsilon_{G}dx,\;\;\;\;TM \end{array}$$

where $\varepsilon_{G}$, $\mu_{G}$, $n_{G}$, $d_{G}$, and $\theta_{G}$ are, respectively, the permittivity, the permeability, the refractive index, the thickness of the gradient-material layer, and the angle between the wave vector and the surface normal vector of the layer. From Equations (8) and (9) and Equation (4), the characteristic matrix of the gradient material can be calculated.

Then, the amplitudes of the incidence, transmitted, and reflected waves for the whole PC structure can be connected by the matrix:

(10) $$M=\overset{N}{\underset{i=1}{\prod }}M_{i}=\left[\begin{array}{cc} m_{11} & m_{12}\\ m_{21} & m_{22} \end{array}\right].$$

And the reflectivity and transmissivity can be derived out to be

(11) $$R={\left|\frac{\left(m_{11}+m_{12}p_{B}\right)p_{A}-\left(m_{21}+m_{22}p_{B}\right)}{\left(m_{11}+m_{12}p_{B}\right)p_{A}+\left(m_{21}+m_{22}p_{B}\right)}\right|}^{2},$$

(12) $$T=\frac{p_{B}}{p_{A}}{\left|\frac{2p_{A}}{\left(m_{11}+m_{12}p_{B}\right)p_{A}+\left(m_{21}+m_{22}p_{B}\right)}\right|}^{2},$$

where $p_{A\left(B\right)}=\sqrt{\varepsilon_{A\left(B\right)}/\mu_{A\left(B\right)}}cos\theta$ for TE wave, and $p_{A\left(B\right)}=\sqrt{\mu_{A\left(B\right)}/\varepsilon_{A\left(B\right)}}cos\theta$ for TM wave, and A and B represent the space at the left and right side of the whole PC structure, with $\varepsilon_{A\left(B\right)}$ and $\mu_{A\left(B\right)}$ being the permittivity and permeability of A or B, respectively. It can be easily obtained that $p_{A}=p_{B}=cos\theta$ when the left and right side of the whole structure are vacuum. It should be noted that Equations (11) and (12) are suitable for the isotropic media [27], however, these two equations are not applicable for the anisotropic media [28,29].

## 3. Numerical Results and Discussions

In the following, in Section 3.1 we first discuss the PBG properties of Models 1 and 2, and then for Models 3 and 4; in Section 3.2 we will study the properties of defect modes in Models 5 and 6.

### 3.1. Research of PBG

#### 3.1.1. PBG Properties of Model 1 ${\left(H^{\prime }L\right)}^{N}$ and Model 2 ${\left(H^{\prime \prime }L\right)}^{N}$

**Figure 2:**
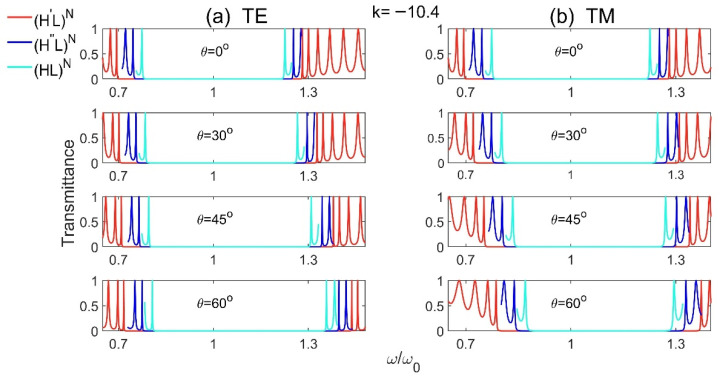
The transmission spectra of Model 1 ${\left(H^{\prime }L\right)}^{N}$, Model 2 ${\left(H^{\prime \prime }L\right)}^{N}$ and the reference model ${\left(HL\right)}^{N}\;$ for different incident angles at slope k=−10.4 of the gradient materials for (**a**) TE and (**b**) TM polarizations.

We firstly discuss the properties of PBG in Model 1 ${\left(H^{\prime }L\right)}^{N}$ and Model 2 ${\left(H^{\prime \prime }L\right)}^{N}$. As mentioned above, Models 1 and 2 represent the complete 1D GPCs with high-index gradient-material layers and low-index normal-material layers, and $H^{\prime }$ and $H^{\prime \prime }$ are high-index gradient materials with first- and second-order (u=1, 2), respectively. The transmission spectra for different incident angles at slope k=−10.4 of the gradient materials are plotted as shown in Figure 2. For comparison, the transmission spectra of reference model ${\left(HL\right)}^{N}$ with normal materials are also studied. The parameters for Figure 2 are selected as: ${\bar{n}}_{H^{\prime }}={\bar{n}}_{H^{\prime \prime }}=n_{H}=3.6$, $n_{L}=1.8$, ${\bar{n}}_{H^{\prime }}d_{H^{\prime }}={\bar{n}}_{H^{\prime \prime }}d_{H^{\prime \prime }}=n_{L}d_{L}=\lambda_{0}/4$, *N* = 15, and we consider all the materials are non-magnetic with μ=1. It should be noted that the slopes k of the high-index gradient materials ($H^{\prime }$ and $H^{\prime \prime }$) should be kept in −10.4~10.4, otherwise the refractive index of the gradient materials may become negative while keeping ${\bar{n}}_{H^{\prime }}={\bar{n}}_{H^{\prime \prime }}=3.6$.

From Figure 2, we see that, no matter for the structures with or without gradient materials, the PBGs become bigger with the increase of incident angle for TE wave, and become smaller for TM wave. However, at a certain incident angle, no matter for TE or TM waves, the PBGs of 1D GPCs are always larger than that of 1D NPCs, and the structure with first-order (u=1) gradient materials has the largest PBG, i.e., the PBGs of ${\left(H^{\prime }L\right)}^{N}$ and ${\left(H^{\prime \prime }L\right)}^{N}$ are always larger than ${\left(HL\right)}^{N}$, and the PBG of ${\left(H^{\prime }L\right)}^{N}$ is the largest one. The 1D GPCs with high-index gradient materials benefit to achieve larger PBG than that in 1D NPCs.

**Figure 3:**
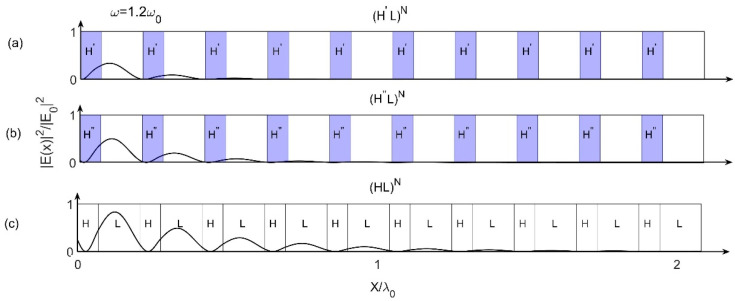
Field distributions for $\omega =1.2\omega_{0}$ and k=−10.4 at incident angle θ=0° for (**a**) Model 1 ${\left(H^{\prime }L\right)}^{N}$, (**b**) Model 2 ${\left(H^{\prime \prime }L\right)}^{N}$ and (**c**) reference model ${\left(HL\right)}^{N}$, respectively.

The above results can be understood by the field distributions as shown in Figure 3, in which the Bragg scattering effect inside these three models can be observed. Figure 3 shows the field distributions of these three models for $\omega =1.2\omega_{0}$ and k=−10.4 at incident angle θ=0°, where all the parameters are the same as those in Figure 2. We know that in field distributions, the lower the peak amplitude of the field is, the stronger the Bragg scattering effect is [30,31]. From Figure 3, we can see that, the peaks of ${\left(H^{\prime }L\right)}^{N}$ and ${\left(H^{\prime \prime }L\right)}^{N}$ with gradient materials are lower than ${\left(HL\right)}^{N}$ with normal materials (we can see clearly from the first peak on the far left), and the peak of ${\left(H^{\prime }L\right)}^{N}$ with first-order gradient material is the lowest. So that the Bragg scattering effects for ${\left(H^{\prime }L\right)}^{N}$ and ${\left(H^{\prime \prime }L\right)}^{N}$ are stronger than that for ${\left(HL\right)}^{N}$, and the effect for ${\left(H^{\prime }L\right)}^{N}$ is the strongest. Since the stronger Bragg scattering effect results in larger PBG, so that the PBGs of ${\left(H^{\prime }L\right)}^{N}$ and ${\left(H^{\prime \prime }L\right)}^{N}$ are larger than that of ${\left(HL\right)}^{N}$, and the PBG of ${\left(H^{\prime }L\right)}^{N}$ is the largest one. It is very consistent with the results obtained from Figure 2.

**Figure 4:**
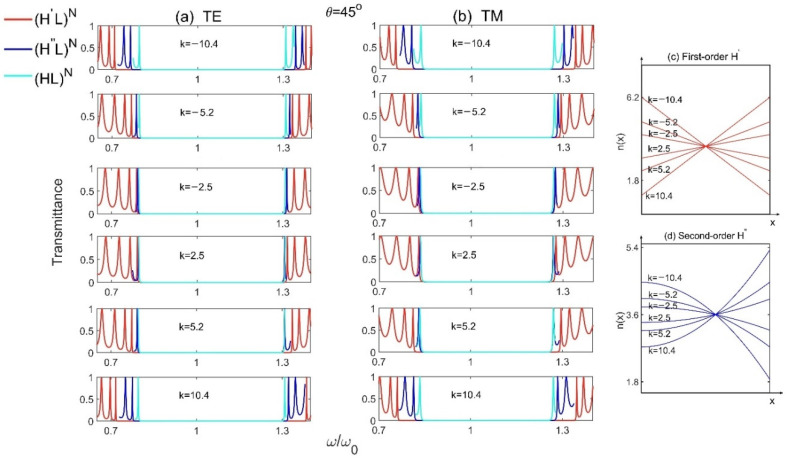
The transmission spectra of Model 1 ${\left(H^{\prime }L\right)}^{N}$, Model 2 ${\left(H^{\prime \prime }L\right)}^{N}$ and the reference Model ${\left(HL\right)}^{N}\;$ for different slopes k at incident angle θ=45° for (**a**) TE and (**b**) TM polarizations, and (**c**,**d**) are the distributions of refractive index for different slopes k for the first- and second-order gradient material $H^{\prime }$ and $H^{\prime \prime }$, respectively.

In order to study the influence of the slope k on PBG, the transmission spectra of Model 1 ${\left(H^{\prime }L\right)}^{N}$, Model 2 ${\left(H^{\prime \prime }L\right)}^{N}$ and the reference Model ${\left(HL\right)}^{N}\;$ for different slopes k at incident angle θ=45° are plotted as shown in Figure 4a,b, where all the parameters are the same as those in Figure 2. From these two figures, we can see that, no matter for TE or TM waves, the PBGs increase with the increase of the absolute value of k. The bigger the absolute value of k is, the larger the PBGs for 1D GPCs will be. And the structure ${\left(H^{\prime }L\right)}^{N}$ with first-order gradient materials still has the largest PBG. Besides, the distributions of refractive index of the high-index gradient-material layers $H^{\prime }$ and $H^{\prime \prime }$ for different slopes k are also presented, as shown in Figure 4c,d. We see that, although the average refractive indexes of the gradient materials are keeping at ${\bar{n}}_{H^{\prime }}={\bar{n}}_{H^{\prime \prime }}=3.6$, the range of variation in indexes increase with the increase of |k|, and the first-order gradient material $H^{\prime }$ possesses more sharp or obvious change in indexes compared with that in the second-order gradient material $H^{\prime \prime }$, resulting stronger Bragg scattering and larger PBG. The changes of k agree well with the changes of PBG as shown in Figure 4a,b.

To further clarify the PBG properties of the models, the PBG, omni-PBG, and complete PBG (C-PBG) for TE and TM waves are listed as shown in Table 1. From Table 1, we can also obtain the conclusions that have been previously obtained from Figure 2 and Figure 4. Besides, we can clearly see the improvements of PBG for 1D GPCs. For example, when k=−5.2, for TE wave the omni-PBGs of ${\left(H^{\prime }L\right)}^{N}$ and ${\left(H^{\prime \prime }L\right)}^{N}$ are 12.7% and 5.7% larger than that in the reference model ${\left(HL\right)}^{N}$; for TM wave the corresponding promotions are 18.3% and 8.4%. When k=−10.4, we achieve the optimal improvements. For TE wave the optimal omni-PBGs are 38.6% and 15.9%, and for TM wave 50.2% and 22.3%, larger than that in the reference model. It is worth noting that all of our comparisons are obtained under the condition of keeping the average refractive indexes of the gradient materials in 1D GPCs the same as the index of the corresponding normal materials in the 1D NPC (the reference model).

**Figure 5:**
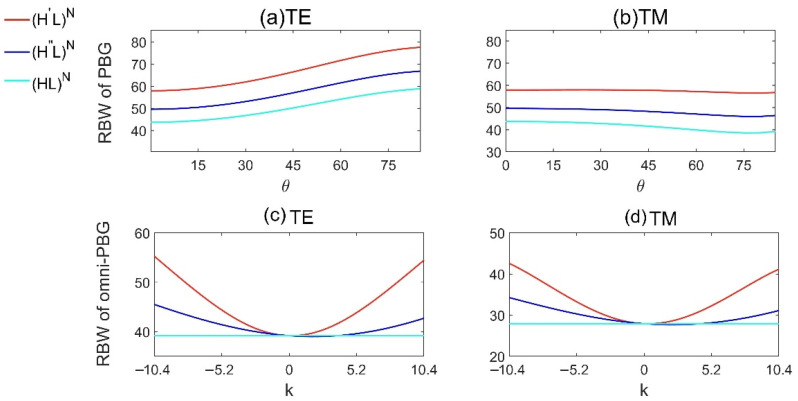
The relative bandwidth of PBG and omni-PBG for Model 1 ${\left(H^{\prime }L\right)}^{N}$, Model 2 ${\left(H^{\prime \prime }L\right)}^{N}$ and the reference model ${\left(HL\right)}^{N}$, (**a**,**b**) are relative bandwidth of PBG for different incident angles at k=−10.4, and (**c**,**d**) are relative bandwidth of omni-PBG for different slopes k.

In addition, we consider the relative bandwidth (RBW, RBW=Bandwidth/Central frequency) of the three models. Figure 5a,b show the RBWs of PBG for different incident angles at k=−10.4, and Figure 5c,d show the RBWs of omni-PBG for different slopes k, where all the parameters are the same as those in Figure 2. From Figure 5a,b, we can see that, for all the three models, the RBWs of PBG become larger for TE wave and smaller for TM wave, with the increase of incident angle. For TE wave, the optimal RBWs of PBG of ${\left(H^{\prime }L\right)}^{N}$ and ${\left(H^{\prime \prime }L\right)}^{N}$ are 32.2% and 12.7% larger than that of the reference model ${\left(HL\right)}^{N}$; for TM wave the corresponding promotions are 31.8% and 13.6%. The RBWs of PBG for 1D GPCs with high-index gradient material are always larger than that in 1D NPCs, and the RBW of PBG for ${\left(H^{\prime }L\right)}^{N}$ is the best. From Figure 5c,d we see that, no matter for TE or TM waves, the RBWs of omni-PBG increase with the increase of the absolute value of k. When k=−10.4, for TE wave the optimal RBWs of omni-PBG of ${\left(H^{\prime }L\right)}^{N}$ and ${\left(H^{\prime \prime }L\right)}^{N}$ are 41.1% and 16.1% larger than that in the reference model ${\left(HL\right)}^{N}$; For TM wave the corresponding promotions are 52.3% and 22.6%. It is obvious that the 1D GPCs with high-index gradient materials benefit to achieve larger omni-PBG than that in 1D NPCs.

#### 3.1.2. PBG Properties of Model 3 ${\left(HL^{\prime }\right)}^{N}$ and Model 4 ${\left(HL^{\prime \prime }\right)}^{N}$

**Figure 6:**
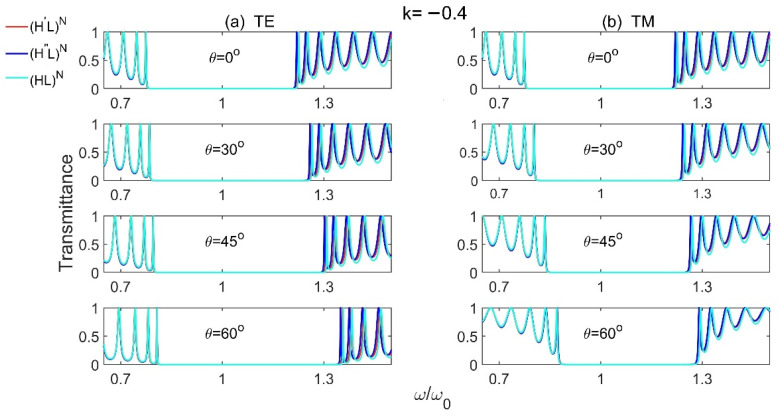
The transmission spectra of Model 3 ${\left(HL^{\prime }\right)}^{N}$, Model 4 ${\left(HL^{\prime \prime }\right)}^{N}$ and the reference model ${\left(HL\right)}^{N}\;$ for different incident angles at slope k=−0.4 of the gradient materials for (**a**) TE and (**b**) TM polarizations.

**Figure 7:**
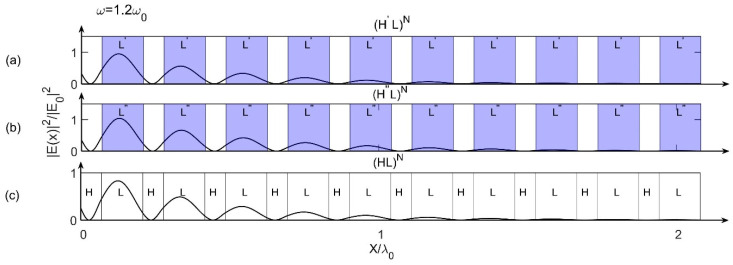
Field distributions for $\omega =1.2\omega_{0}$ and k=−0.4 at incident angle θ=0° for (**a**) Model 3 ${\left(HL^{\prime }\right)}^{N}$, (**b**) Model 4 ${\left(HL^{\prime \prime }\right)}^{N}$ and (**c**) reference Model ${\left(HL\right)}^{N}$, respectively.

**Figure 8:**
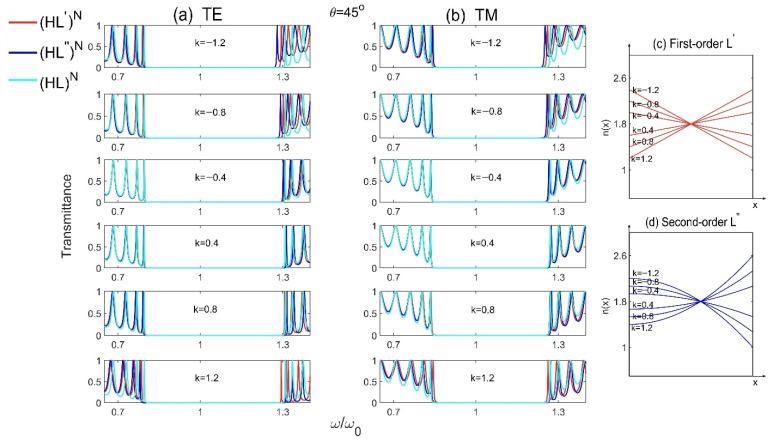
The transmission spectra of Model 3 ${\left(HL^{\prime }\right)}^{N}$, Model 4 ${\left(HL^{\prime \prime }\right)}^{N}$ and the reference Model ${\left(HL\right)}^{N}\;$ for different slopes k at incident angle θ=45° for (**a**) TE and (**b**) TM polarizations, and (**c**,**d**) are the distributions of refractive index for different slopes k for the first- and second-order gradient material $L^{\prime }$ and $L^{\prime \prime }$, respectively.

Then, we discuss the PBG properties of Model 3 ${\left(HL^{\prime }\right)}^{N}$ and Model 4 ${\left(HL^{\prime \prime }\right)}^{N}$, which represent the complete 1D GPCs with high-index normal-material layers and low-index gradient-material layers. $L^{\prime }$ and $L^{\prime \prime }$ are low-index gradient materials with first- and second-order (u=1, 2), respectively. Similarly, the transmission spectra for different incident angles at the same slope, the field distributions for these three models, and the transmission spectra for different slopes k at the same incident angle θ=45°, are shown in Figure 6, Figure 7 and Figure 8, respectively, where all the parameters are selected as: $n_{H}=3.6$,$\;{\bar{n}}_{L^{\prime }}={\bar{n}}_{L^{\prime \prime }}=n_{L}=1.8$, $\;{\bar{n}}_{H}d_{H}={\bar{n}}_{L^{\prime }}\;d_{L^{\prime }}=n_{L^{\prime \prime }}d_{L^{\prime \prime }}=\lambda_{0}/4$, *N* = 15, and all the materials are non-magnetic with μ=1. It should be noted that the slopes k of the low-index gradient materials ($L^{\prime }$ and $L^{\prime \prime }$) cannot be too large and are kept in −1.2~1.6, otherwise the refractive index of the gradient materials may become negative while keeping ${\bar{n}}_{L^{\prime }}={\bar{n}}_{L^{\prime \prime }}=1.8$.

From Figure 6, we can find that in these three models, the PBGs still become bigger with the increase of incident angle for TE wave, and become smaller for TM wave. However, at a certain incident angle, the PBGs are almost the same for these three models. The field distributions in Figure 7 show that the peaks for these three models have no obvious difference, which agree well with the performances of PBGs obtained from Figure 6. From Figure 8, we see that the PBGs have only a little change with the increase of |k| for both TE and TM waves. The situations are quite different from those in model 1 ${\left(H^{\prime }L\right)}^{N}$ and Model 2 ${\left(H^{\prime \prime }L\right)}^{N}$. As shown in Figure 8c,d, the range of variation in indexes for $L^{\prime }$ and $L^{\prime \prime }$ in Model 3 ${\left(HL^{\prime }\right)}^{N}$ and Model 4 ${\left(HL^{\prime \prime }\right)}^{N}$ are not as strong as $H^{\prime }\;$and $H^{\prime \prime }$ in Model 1 ${\left(H^{\prime }L\right)}^{N}$ and Model 2 ${\left(H^{\prime \prime }L\right)}^{N}$, so that the changes of PBG are not obvious. The improvements of RBW in these three models are also not obvious, so here we omit the figures of RBW. Thus, the complete 1D GPCs with low-index gradient materials may not benefit to achieve larger PBG than that in 1D NPCs.

### 3.2. Research of Defect Mode in Model 5 ${\left(HL\right)}^{N}D^{\prime }{\left(LH\right)}^{N}$ and Model 6 ${\left(HL\right)}^{N}D^{\prime \prime }{\left(LH\right)}^{N}$

Next, we will discuss the defect mode properties of Model 5 ${\left(HL\right)}^{N}D^{\prime }{\left(LH\right)}^{N}$ and Model 6 ${\left(HL\right)}^{N}D^{\prime \prime }{\left(LH\right)}^{N}$, in which $D^{\prime }$ and $D^{\prime \prime }$ represent the gradient defects with first- and second-order, respectively. For comparison, the defect mode properties of ${\left(HL\right)}^{N}D{\left(LH\right)}^{N}$ with normal-material defect are also studied. In the following, the parameters for these three models are selected as: $n_{H}=3.6$, $n_{L}=1.8$, ${\bar{n}}_{D^{\prime }}={\bar{n}}_{D^{\prime \prime }}=n_{D}=4.5$, $n_{H}d_{H}=n_{L}d_{L}=\lambda_{0}/4$, ${\bar{n}}_{D^{\prime }}d_{D^{\prime }}={\bar{n}}_{D^{\prime \prime }}d_{D^{\prime \prime }}=n_{D}d_{D}=\lambda_{0}/2$, N=15, and all the materials have μ=1.

**Figure 9:**
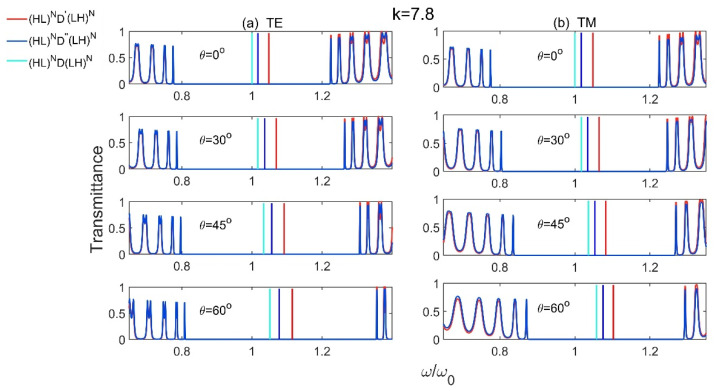
The transmission spectra of Model 5 ${\left(HL\right)}^{N}D^{\prime }{\left(LH\right)}^{N}$, Model 6 ${\left(HL\right)}^{N}D^{\prime \prime }{\left(LH\right)}^{N}$ and the reference Model ${\left(HL\right)}^{N}D{\left(LH\right)}^{N}\;$ for different incident angles at slope k=7.8 of the gradient defects for (**a**) TE and (**b**) TM polarizations.

Figure 9 shows the defect mode properties for different incident angles at slope k=7.8 of the gradient defects in Model 5 ${\left(HL\right)}^{N}D^{\prime }{\left(LH\right)}^{N}$, Model 6 ${\left(HL\right)}^{N}D^{\prime \prime }{\left(LH\right)}^{N}$, and the reference model ${\left(HL\right)}^{N}D{\left(LH\right)}^{N}$. From Figure 9, we can see that, no matter for TE or TM waves, the defect modes move to the high-frequency region (blue shift) with the increase of the incident angles. However, the defect modes for structures with gradient defects shift faster to the high-frequency region than that with normal material defect, and the defect modes for structure with first-order (u=1) gradient defect have the fastest shift.

**Figure 10:**
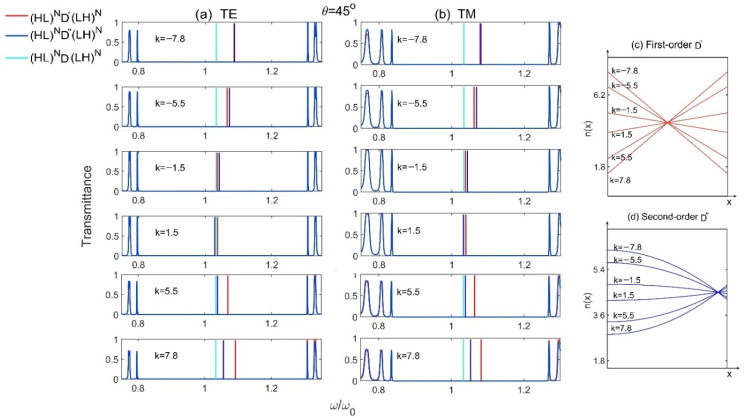
The transmission spectra of Model 5 ${\left(HL\right)}^{N}D^{\prime }{\left(LH\right)}^{N}$, Model 6 ${\left(HL\right)}^{N}D^{\prime \prime }{\left(LH\right)}^{N}$ and the reference Model ${\left(HL\right)}^{N}D{\left(LH\right)}^{N}\;$ for different slopes k at incident angle θ=45° for (**a**) TE and (**b**) TM polarizations, and (**c**,**d**) are the distributions of refractive index for different slopes k for the first- and second-order gradient defect $D^{\prime }$ and $D^{\prime \prime }$, respectively.

Furthermore, the transmission spectra and the refractive index distributions for different slopes k are plotted as shown in Figure 10, where all the parameters are the same as those in Figure 9. We can find that, no matter for TE or TM waves, the frequencies of defect modes decrease with the decrease of the absolute value of k. And the changes of defect modes are asymmetrical with respect to the value of k since the changes of refractive index for the gradient defects are not symmetrical with the change of k as shown in Figure 10c,d.

To clearly show the defect mode properties of the models, the frequencies of defect mode for different slopes of gradient defect and incident angles for all the three models are listed as shown in Table 2, from which we can clearly see the changes of defect modes for different situations. For example, when k=7.8, for TE wave the frequencies of defect modes are 4.79% and 1.69% for model 5 ${\left(HL\right)}^{N}D^{\prime }{\left(LH\right)}^{N}$ and model 6 ${\left(HL\right)}^{N}D^{\prime \prime }{\left(LH\right)}^{N}$ higher than that for the reference model ${\left(HL\right)}^{N}D{\left(LH\right)}^{N}$ at θ=0°, 5.64% and 2.21% higher at θ=45°, and 6.08% and 2.51% higher at θ=60°; for TM wave the frequencies of defect modes are 4.79% and 1.69% higher at θ=0°, 4.48% and 1.69% higher at θ=45°, and 4.25% and 1.65% higher at θ=60°. With the increase of incident angle, the defect modes for both Model 5 and Model 6 shift faster to the high-frequency region than that in the reference model. It should be pointed out that all of our comparisons are obtained under the condition of keeping the average refractive indexes of the gradient defects in 1D GPCs the same as the index of the corresponding normal material defect in the 1D NPCs (the reference Model).

**Figure 11:**
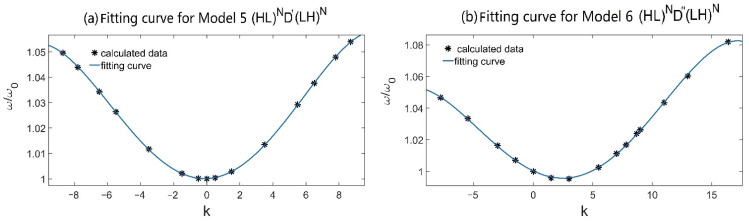
The fitting curves for frequencies of defect modes and slopes k of the gradient defects at incident angle θ=0° for (**a**) Model 5 ${\left(HL\right)}^{N}D^{\prime }{\left(LH\right)}^{N}$ and (**b**) Model 6 ${\left(HL\right)}^{N}D^{\prime \prime }{\left(LH\right)}^{N}$.

In addition, by using the fitting curves method, we obtain the relationship between frequencies of defect modes and slopes k of the gradient defects. Figure 11 shows the fitting curves for both of the models at incident angle θ=0°, where the fitting curve equations can be obtained as $\omega_{D^{\prime }}=1+2.573\times 10^{-4}k+1.067\times 10^{-3}k^{2}-3.683\times 10^{-8}k^{3}-5.107\times 10^{-6}k^{4}$ for Model 5 ${\left(HL\right)}^{N}D^{\prime }{\left(LH\right)}^{N}$ and $\omega_{D^{\prime \prime }}=1-3.560\times 10^{-3}k+6.406\times 10^{-4}k^{2}+2.633\times 10^{-5}k^{3}-2.057\times 10^{-6}k^{4}$ for Model 6 ${\left(HL\right)}^{N}D^{\prime \prime }{\left(LH\right)}^{N}$, respectively. In this way, one can hope to obtain the needed defect mode by selecting a proper slope k of the gradient material, which is useful for designing tunable narrow-band PC filters.

## 4. Conclusions

In summary, we have studied the transmission properties of 1D PCs composed of gradient materials. By using the TMM method, we find that the complete 1D GPCs with high-index gradient materials benefit to achieve larger omni-PBG than that in 1D NPCs. In our high-index gradient materials case, for TE(TM) wave, the optimal omni-PBGs for 1D GPCs with first- and second-order gradient materials are 38.6% (50.2%) and 15.9% (22.3%) larger than that in 1D NPCs; while for the optimal relative bandwidths of omni-PBG, the corresponding promotions are 41.1% (52.3%) and 16.1% (22.6%), respectively. The position of defect modes of 1D GPCs with gradient defect can be adjusted by selecting proper parameters of the gradient materials. It should be pointed out that, in our study, when comparing the performances of PCs, the average refractive index of the gradient materials in the 1D GPCs are always kept the same as the index of the corresponding normal materials in the 1D NPCs (the reference model). It should be noted that the results in this paper have been checked with the finite element method by using the commercial software COMSOL. And the results for the two methods fit quite well. These researches may provide a promising way for designing wide PBG devices and tunable narrow-band filters in optical communication.

## Figures and Tables

**Table 1:** PBG, omni-PBG, C-PBG (in unit $\omega /\omega_{0}$ ) for Model 1 ${\left(H^{\prime }L\right)}^{N}$, Model 2 ${\left(H^{\prime \prime }L\right)}^{N}$ and the reference model ${\left(HL\right)}^{N}$.

		TE			TM		
	θ	${\left(H^{\prime }L\right)}^{N}$	${\left(H^{\prime \prime }L\right)}^{N}$	${\left(HL\right)}^{N}$	${\left(H^{\prime }L\right)}^{N}$	${\left(H^{\prime \prime }L\right)}^{N}$	${\left(HL\right)}^{N}$
k=−10.4	0°	0.5792	0.4958	0.4370	0.5792	0.4958	0.4370
	30°	0.6192	0.5307	0.4676	0.5796	0.4904	0.4279
	45°	0.6645	0.5701	0.5020	0.5776	0.4824	0.4155
	60°	0.7164	0.6152	0.5413	0.5723	0.4705	0.3988
	Omni-PBG	0.5533	0.4626	0.3992	0.4485	0.3650	0.2986
	C-PBG	0.4485	0.3650	0.2986	The same as TE case		
k=−5.2	0°	0.4835	0.4583	0.4370	0.4835	0.4583	0.4370
	30°	0.5173	0.4904	0.4676	0.4778	0.4505	0.4279
	45°	0.5554	0.5268	0.5020	0.4694	0.4396	0.4155
	60°	0.5989	0.5683	0.5413	0.4572	0.4247	0.3988
	Omni-PBG	0.4500	0.4220	0.3992	0.3532	0.3236	0.2986
	C-PBG	0.3532	0.3236	0.2986	The same as TE case		
k=−2.5	0°	0.4491	0.4446	0.4370	0.4491	0.4446	0.4370
	30°	0.4805	0.4757	0.4676	0.4409	0.4359	0.4279
	45°	0.5158	0.5109	0.5020	0.4296	0.4240	0.4155
	60°	0.5562	0.5510	0.5413	0.4140	0.4079	0.3988
	Omni-PBG	0.4125	0.4074	0.3992	0.3138	0.3078	0.2986
	C-PBG	0.3138	0.3078	0.2986	The same as TE case		
k=2.5	0°	0.4475	0.4352	0.4370	0.4475	0.4352	0.4370
	30°	0.4788	0.4655	0.4676	0.4392	0.4260	0.4279
	45°	0.5142	0.4996	0.5020	0.4280	0.4137	0.4155
	60°	0.5546	0.5386	0.5413	0.4128	0.3972	0.3988
	Omni-PBG	0.4101	0.3971	0.3992	0.3088	0.2958	0.2986
	C-PBG	0.3088	0.2958	0.2986	The same as TE case		
k=5.2	0°	0.4808	0.4399	0.4370	0.4808	0.4399	0.4370
	30°	0.5146	0.4705	0.4676	0.4751	0.4313	0.4279
	45°	0.5528	0.5049	0.5020	0.4668	0.4197	0.4155
	60°	0.5965	0.5443	0.5413	0.4552	0.4042	0.3988
	Omni-PBG	0.4460	0.4024	0.3992	0.3445	0.3004	0.2986
	C-PBG	0.3445	0.3004	0.2986	The same as TE case		
k=10.4	0°	0.5762	0.4677	0.4370	0.5762	0.4677	0.4370
	30°	0.6165	0.5002	0.4676	0.5768	0.4617	0.4279
	45°	0.6623	0.5368	0.5020	0.5753	0.4532	0.4155
	60°	0.7148	0.5787	0.5413	0.5706	0.4415	0.3988
	Omni-PBG	0.5487	0.4327	0.3992	0.4375	0.3310	0.2986
	C-PBG	0.4375	0.3310	0.2986	The same as TE case		

**Table 2:** Defect mode frequencies (in unit $\omega /\omega_{0}$ ) of Model 5 ${\left(HL\right)}^{N}D^{\prime }{\left(LH\right)}^{N}$, Model 6 ${\left(HL\right)}^{N}D^{\prime \prime }{\left(LH\right)}^{N}$ and the reference model ${\left(HL\right)}^{N}D{\left(LH\right)}^{N}$ for ${\bar{n}}_{D^{\prime }}={\bar{n}}_{D^{\prime \prime }}=4.5$.

		TE			TM		
	θ	${\left(HL\right)}^{N}D^{\prime }{\left(LH\right)}^{N}$	${\left(HL\right)}^{N}D^{\prime \prime }{\left(LH\right)}^{N}$	${\left(HL\right)}^{N}D{\left(LH\right)}^{N}$	${\left(HL\right)}^{N}D^{\prime }{\left(LH\right)}^{N}$	${\left(HL\right)}^{N}D^{\prime \prime }{\left(LH\right)}^{N}\;\;$	${\left(HL\right)}^{N}D{\left(LH\right)}^{N}$
k=−7.8	0°	1.0439	1.0466	1	1.0439	1.0466	1
	30°	1.0650	1.0672	1.0165	1.0603	1.0628	1.0171
	45°	1.0869	1.0884	1.0335	1.0783	1.0805	1.0361
	60°	1.1095	1.1102	1.0510	1.0982	1.1003	1.0576
k=−5.5	0°	1.0264	1.0334	1	1.0264	1.0334	1
	30°	1.0458	1.0528	1.0165	1.0432	1.0500	1.0171
	45°	1.0658	1.0729	1.0335	1.0617	1.0683	1.0361
	60°	1.0866	1.0937	1.0510	1.0824	1.0887	1.0576
k=−1.5	0°	1.0021	1.0071	1	1.0021	1.0071	1
	30°	1.0189	1.0242	1.0165	1.0192	1.0241	1.0171
	45°	1.0361	1.0418	1.0335	1.0382	1.0430	1.0361
	60°	1.0539	1.0599	1.0510	1.0596	1.0643	1.0576
k=1.5	0°	1.0028	0.9959	1	1.0028	0.9959	1
	30°	1.0197	1.0122	1.0165	1.0199	1.0131	1.0171
	45°	1.0370	1.0290	1.0335	1.0389	1.0322	1.0361
	60°	1.0548	1.0463	1.0510	1.0603	1.0538	1.0576
k=5.5	0°	1.0292	1.0026	1	1.0292	1.0026	1
	30°	1.0489	1.0201	1.0165	1.0461	1.0200	1.0171
	45°	1.0692	1.0383	1.0335	1.0647	1.0392	1.0361
	60°	1.0903	1.0571	1.0510	1.0854	1.0608	1.0576
k=7.8	0°	1.0479	1.0169	1	1.0479	1.0169	1
	30°	1.0694	1.0362	1.0165	1.0644	1.0343	1.0171
	45°	1.0918	1.0564	1.0335	1.0825	1.0536	1.0361
	60°	1.1149	1.0774	1.0510	1.1025	1.0750	1.0576

## Data Availability

The data are contained within the article. Additional data are available upon request from the corresponding author.
